# Potential Impact of Polymorphisms in Toll-like Receptors 2, 3, 4, 7, 9, miR-146a, miR-155, and miR-196a Genes on Osteoarthritis Susceptibility

**DOI:** 10.3390/biology12030458

**Published:** 2023-03-16

**Authors:** Debora Stefik, Vladimir Vranic, Nemanja Ivkovic, Gordana Velikic, Dusan M. Maric, Dzihan Abazovic, Danilo Vojvodic, Dusica L. Maric, Gordana Supic

**Affiliations:** 1 Institute for Medical Research, Military Medical Academy, Crnotravska 17, 11000 Belgrade, Serbia; 2 Clinic for Orthopedic Surgery and Traumatology, Military Medical Academy, Crnotravska 17, 11000 Belgrade, Serbia; 3 Medical Faculty of Military Medical Academy, University of Defense, Crnotravska 17, 11000 Belgrade, Serbia; 4 Department for Research and Development, Clinic Orto MD-Parks Dr Dragi Hospital, 21000 Novi Sad, Serbia; 5 Biocell Hospital, Omladinskih Brigada 86a, 11000 Belgrade, Serbia; 6 Department of Anatomy, Faculty of Medicine, University of Novi Sad, 21000 Novi Sad, Serbia

**Keywords:** Toll-like receptors, polymorphisms, TLR4, TLR7, microRNA, miR-196a, osteoarthritis

## Abstract

**Simple Summary:**

Osteoarthritis (OA) is a progressive inflammatory disease and a leading cause of disability among elders. Accumulating evidence suggests that inflammation-related genes, including genes for Toll-like receptors (TLRs), could play an important role in the susceptibility to and pathogenesis of OA. Toll-like receptors are controlled by several microRNAs, which in addition to their role in the epigenetic regulation of gene expression on a post-transcriptional level, are ligands for TLR activation and downstream signaling. Thus, we evaluated the association between OA risk and genetic variants in TLR2, TLR3, TLR4, TLR7, TLR9, and microRNAs that regulate TLR signaling miR-146a, miR-155, and miR-196a2. Our findings indicate that polymorphisms in the TLR4 and TLR7 genes could increase OA risk, and shows a novel suggestive association of the miR-196a2 polymorphism rs11614913 variant allele with a decreased susceptibility to OA. The modulation of TLRs and miRNAs and their cross-talk might be an attractive target for a personalized approach to OA management.

**Abstract:**

Osteoarthritis (OA) is a progressive inflammatory disease of synovial joints and a leading cause of disability among adults. Inflammation-related genes, including genes for Toll-like receptors (TLRs), are tightly controlled by several microRNAs that, in addition to their pivotal role in the epigenetic regulation of target genes, are ligands for TLR activation and downstream signaling. Thus, we evaluated the association between OA risk and genetic variants in TLR2, TLR3, TLR4, TLR7, TLR9, and microRNAs that regulate TLRs signaling miR146a, miR155, and miR196a2. Our study group consisted of 95 surgically treated OA patients and a control group of 104 healthy individuals. Genetic polymorphisms were determined using TaqMan real-time PCR assays (Applied Biosystems). Adjusted logistic regression analysis demonstrated that polymorphisms in TLR4 rs4986790 (OR = 2.964, *p* = 0.006), TLR4 rs4986791 (OR = 8.766, *p* = 0.00001), and TLR7 rs385389 (OR = 1.579, *p* = 0.012) increased OA risk, while miR-196a2 rs11614913 (OR = 0.619, *p* = 0.034) was significantly associated with decreased OA risk. Our findings indicate that polymorphisms in the TLR4 and TLR7 genes might increase OA risk and suggest a novel association of miR-196a2 polymorphism with decreased OA susceptibility. The modulation of TLRs and miRNAs and their cross-talk might be an attractive target for a personalized approach to OA management.

## 1. Introduction

Osteoarthritis (OA) is a chronic and progressive disease of the synovial joints characterized by degenerative changes in the articular cartilage, subchondral bone, and supporting musculature [1]. OA is recognized as a disease of major socioeconomic importance because of its frequency and significant impact on quality of life. Systematic reviews of worldwide incidence and prevalence show that 9.6% of men and 18% of women in the population older than 60 years have symptomatic OA [2]. Weight-bearing hip or knee joints are mainly affected by OA, but non-weight-bearing (hand) joints can also be affected. Chronic, low-grade inflammation is triggered by various risk factors such as advanced age, injuries and joint overuse, or obesity [1,2]. The multi-etiologic origin of this disease implies a complex interaction between genetic, immunologic, and metabolic factors. However, the exact molecular mechanisms that trigger pathological responses and contribute to OA progression remain unknown.

Increasing evidence indicates that the innate response pathways could play a critical role in the initiation and progression of OA, in particular Toll-like receptors (TLRs) [3,4,5]. Toll-like receptors are transmembrane proteins with a pivotal role in the pathogen recognition and induction of inflammatory signaling pathways by regulating the activation of antigen-presenting cells and key cytokines, linking innate to adaptive immunity. These receptors initiate a protective immune response not only in response to the presence of pathogens by sensing conserved molecular patterns (pathogen-associated molecular patterns, PAMPs) but also by recognizing specific endogenous host molecules released upon cellular stress and tissue damage (damage-associated molecular patterns, DAMPs). Aberrant activation of TLR contributes to the development of a number of diseases in which pathogenesis inflammation plays a central role [4]. Stimulation of TLRs by different PAMPs/DAMPs initiates signaling cascades that lead to activation of transcription factors such as nuclear factor kappa-light-chain-enhancer of activated B cells (NF-κB), phosphoinositide 3-kinase/protein kinase B (PI3K/AKT), and mitogen-activated protein kinase (MAPK), resulting in a variety of cellular responses, including the production of pro-inflammatory cytokines [4]. In OA, stimulation of TLRs leads to a pro-inflammatory catabolic state that consequently leads to alterations in the anatomic and physiologic functions of the entire joint structure [5]. Once the pro-catabolic state is initiated, more and more pro-inflammatory components are generated, triggering the self-reinforcing cycle of cellular degradation and chronic inflammation.

Accumulated evidence indicates that specific patterns of both innate and adaptive immunity are regulated by epigenetic mechanisms, modifications affecting gene expression that do not involve changing the DNA sequence. In recent years, it has become evident that microRNAs (miRNAs, miRs) play a pivotal role in the epigenetic regulation of target genes. MicroRNAs represent highly conserved small non-coding RNAs, approximately 22 nt in length, that regulate the expression of multiple specific genes on a post-transcriptional level in various cellular processes including cell proliferation, inflammation, apoptosis, and autoimmunity [6]. MicroRNAs are thought to contribute to the development and progression of OA and one of the potential mechanisms could be through their modulatory role in the complex regulatory network of the immune response and TLR-signaling pathways [7]. MiR-146a and miR-155 are among the first-identified and most-studied microRNAs for their multiple roles in the innate and adaptive immune processes, as well as various malignancies [8]. The expression of miR-146a, miR-155, and miR-196a has been associated with various chronic inflammatory and autoimmune diseases, such as rheumatoid arthritis (RA), systemic lupus erythematosus (SLE), type 1 diabetes, periodontitis, atherosclerosis, and inflammatory bowel disease [8,9,10]. Integrative molecular profiling of RNA-sequencing data and analysis of signaling pathways in OA revealed that miR-196a, miR-146a, and miR-155 are differentially expressed between normal and OA cartilage [11]. Several inflammatory stimuli, such as cytokines Tumor necrosis factor-α (TNFα), Interleukin-1 β (IL-1β), and Interferons (IFNs), as well as TLR ligands, induce the expression of miR-146a and miR-155, which subsequently target several TLR4 effectors, such as Suppressor of cytokine signaling 1 (SOCS1), TNFR-associated factor 6 (TRAF6), IL-1R-associated kinase 1 (IRAK1), IRAK2, Interferon regulatory factor 3 (IRF3), and IRF5 [8,12].

Recent studies indicate that polymorphisms in the TLR and microRNA genes could predispose one to various conditions characterized by chronic inflammation, including OA. The rs5743708 polymorphism in exon 3 of the TLR2 gene (Arg753Gln, R753Q) has a negative impact on TLR2 function [13]. It has been shown that two polymorphisms in the TLR3 gene, rs3775291 located in exon 4 (G13909A, L412F) and rs5743312 (C9948T) located in the intron 3 region, result in a change in the structure of the ligand-binding domain on the surface of the TLR3 receptor, affecting its activity [14]. The polymorphism rs4986790 in exon 3 of the TLR4 gene causes an A896G (D299G) substitution, while rs986791 in exon 4 causes a C1196T (T399I), and both result in structural changes in the TLR4 extracellular domain [15]. The polymorphism rs3853839 (C/G) located in the 3′ untranslated region (UTR) of the TLR7 gene is associated with upregulated mRNA expression and increased SLE risk [16], and, recently, with knee osteoarthritis susceptibility [17]. Polymorphism rs187084 (A/G), located in the promoter of the TLR9 gene, alters TLR9 expression and previously was associated with hip OA risk [18]. Two of the most-studied polymorphisms in miRNA genes, miR-146a rs2910164 (C/G) and miR-155 rs767649 (A/T), located in the regulatory regions, have been associated with SLE risk [19]. Polymorphism miR-196a2 rs11614913 (C/T) in the 3′-UTR affects the transcription of miR-196a, which targets gene expression of numerous biological pathways and autoimmune and inflammatory diseases [9,20].

Thus, we aimed to examine associations between common polymorphisms in TLR signaling pathway genes and associated micro RNAs as potential disease-predisposing genetic factors in OA. Our study investigated ten different single-nucleotide polymorphisms (SNPs) in TLR genes (TLR2 rs5743708, TLR3 rs3775291, and rs5743312, TLR4 rs4986790 and rs4986791, TLR7 rs385389, TLR9 rs187084), as well as polymorphisms in miRNA genes (miR-196-a2 rs11614913, miR-146a rs2910164, and miR-155 rs767649) and their association with OA risk.

## 2. Materials and Methods

### 2.1. Study Participants and Biological Samples

A total of 95 patients with radiographically confirmed primary OA that had undergone total hip or knee joint replacement were recruited from the Clinic for Orthopedic Surgery and Traumatology, Military Medical Academy, Belgrade, Serbia. The control group consisted of 104 healthy individuals. Of the total number of 95 patients, 61 patients (64%) had total hip replacement surgery, while 34 patients (36%) had total knee replacement surgery at the Clinic for Orthopedic Surgery and Traumatology, in the period from 2015 to 2018. Excluding criteria for participation in this study were secondary OA due to trauma or joint structure surgeries, congenital malformations, syndromes or congenital hip dysplasia, developmental or hormone/metabolic disorders, infections, gout, and rheumatoid arthritis. In the control group of 104 healthy individuals, the exclusion criteria were OA or other systemic inflammatory or autoimmune diseases and a history of malignancy. The control group and patient group were matched by age and sex, and all participants were Caucasians of the same ethnicity.

### 2.2. Biological Samples, DNA Isolation, and Polymorphism Genotyping

Peripheral blood samples from both OA patients and the control group subjects were collected in tubes and stored at −20 °C until DNA extraction. Genomic DNA for genotype analysis was extracted using the Extract Me Blood Kit according to the manufacturer’s instructions (Blirt, Gdansk, Poland). A 7500 real-time PCR system and the commercially available TaqMan SNP Genotyping Assays (Applied Biosystems, Foster City, CA, USA) were used for the genotype analysis. Gene variants were determined in a reaction volume of 20 µL, composed of 2 × Universal Master Mix (Applied Biosystems), 1.5 µL of genomic DNA, and 40× TaqMan SNP Genotyping Assays, TLR2 (rs5743708, TaqMan Assay ID C_27860663_10), TLR3 (rs3775291, TaqMan Assay ID C_1731425_10; rs5743312. TaqMan Assay ID C_447407_10), TLR4 (rs4986790, TaqMan Assay ID C_11722238_20; rs4986791, TaqMan Assay ID C_117222237_20), TLR7 (rs3853839, TaqMan Assay ID C_2259573_10), TLR9 (rs187084, TaqMan Assay ID C_2301952_10), miR-196a2 (rs11614913, TaqMan Assay ID C_31185852_10), miR-146a (rs2910164, TaqMan Assay ID C_15946974_10), and miR-155 (rs767649, TaqMan Assay ID C_2212229_10). The characteristics of the genotyped TLR and miRNA polymorphisms are presented in Table 1. Real-time amplification was performed under the following conditions: denaturation at 95 °C for 10 min, followed by 40 cycles of denaturation at 95 °C for 15 s and annealing/extension at 60 °C for 60 s. Allelic discrimination was performed using SDS software (v.2.3).

### 2.3. Statistical Analysis

Statistical analysis was performed using SPSS software (version 20.00, SPSS Ins., Chicago, IL, USA). The differences in the genotype frequencies between the patient cohort and control group, as well as the association of TLR and microRNA gene polymorphisms with demographic and etiological data, were calculated using non-parametric Chi-square (χ^2^) or Fisher exact tests. Crude and adjusted odds ratios with 95% confidence intervals were calculated via unconditional binary logistic regression. The association between TLR and miR polymorphisms with osteoarthritis susceptibility was assessed using unconditional logistic regression analysis. Odds ratios (OR) and 95% confidence intervals (95% CI) were calculated and adjusted for age and sex as the potential confounding factors. The recessive genetic model (wild type (wt) genotype vs. combined heterozygous and mutated genotype) as well as the dominant model (combined wt and heterozygous vs. mutated genotype) and additive genetic models of the analyzed gene variants were included in the calculation of OA risk. *p* values of less than 0.05 were considered statistically significant.

## 3. Results

Our case–control study comprised independent 95 osteoarthritis (OA) cases and 104 age- and sex-matched healthy controls, Caucasian of the Serbian population. All of the patients with end-stage OA recruited into the study had total joint arthroplasty (replacement), all fulfilling the inclusion criteria of symptomatic and radiographically confirmed OA. In the group of OA patients, 35 subjects were male and 60 were female, with a median age of 69 years, ranging from 36 to 90 years. Early menopause was present in 27% of patients (15 out of 56 females). OA-relevant clinical characteristics (sex, age, BMI, previous injury, family history, physical activity, smoking, menopause, and early onset of OA) were monitored. The majority of patients were females, with BMI within the overweight range and OA of the hip (64%), while 36% of patients were diagnosed with knee OA. There was no association between TLR gene polymorphisms and demographic and clinicopathological features of OA patients, including Table 2.

We observed an association of the miR-196a2 rs11614913 polymorphism with sex, where the variant allele was associated with the female sex (*p* = 0.014), Table 3. The minor allele of the miR-196a2 rs11614913 polymorphism demonstrated a statistically significant association with a previous history of injury, whereas the variant allele was more frequent in OA patients who did not have a previous injury history (*p* = 0.014). In addition, we observed a tendency towards an association of higher body mass index (BMI) (*p* = 0.051) with miR-196a2 rs11614913 SNP. In our OA cohort, polymorphism rs767649 in the miR-155 gene was associated with early menopause (*p* = 0.036) and exhibited a trend toward an association with sex (*p* = 0.086).

The genotype distributions of the OA patient cohort and healthy controls for the analyzed TLRs and miRNAs genes are shown in Table 4. Our results reveal a significant difference in genotype distributions between OA subjects and controls for both analyzed TLR4 gene polymorphisms rs4986790 and rs4986791 (*p* = 0.004, *p* = 0.0001, respectively). A statistically significant difference in genotype distribution among OA cases and controls was also observed for TLR7 gene variant rs3853839 (*p* = 0.033). Furthermore, a statistically significant difference in genotype distribution was found for miR-196a2 gene polymorphism rs11614913 (*p* = 0.010). Comparison between OA cases and controls did not reveal differences in genotype frequencies for TLR2 rs5743708, TLR3 rs3775291, TLR3 rs5743312, and TLR9 rs187084 polymorphisms, Table 4.

The association between the genotypes of TLRs/miRNAs genes and OA risk was investigated via logistic regression analysis, adjusted for age and sex. As reported in Table 4, TLR4 polymorphisms rs4986790 and rs4986791 were found to be significantly associated with an increased OA risk (OR = 2.964, *p* = 0.006, and OR = 8.766, *p* = 0.00001, respectively), as well as TLR7 rs3853839 (OR = 1.579, *p* = 0.012). Moreover, our present study reveals, for the first time, a significant association of the miR-196a2 rs11614913 polymorphism with decreased OA risk (OR = 0.619, *p* = 0.034), Table 4. Polymorphisms TLR2 rs5743708, TLR3 rs3775291, TLR3 rs5743312, and TLR9 rs187084, as well as miR-146a rs2910164 and miR-155 rs767649 genes are not associated with OA susceptibility; see Table 4.

## 4. Discussion

Inflammation is an essential molecular process in the development and progression of osteoarthritis (OA) [1,5]. Several genome-wide association studies (GWAS) have been conducted to reveal the genetic background of OA susceptibility and inflammation-related genes have been identified among potential genetic variants [5,21,22]. Accumulating evidence suggests that Toll-like receptors (TLRs) may play an important role in the susceptibility, induction, and pathogenesis of numerous diseases whose background is based on immune-mediated processes. Moreover, it is now recognized that OA etiology has an important immunological component, with TLR signaling as the potential key factor in the initiation and maintenance of OA. Toll-like receptors are tightly controlled by several miRNAs, which are ligands for TLR activation and downstream signaling, in addition to their pivotal role in the epigenetic regulation of target genes [12].

Our study found that TLR4 D299G/T399I and TLR7 rs3853839 genetic polymorphisms are associated with an increased OA risk. Furthermore, we revealed a novel suggestive association of miR-196a2 gene polymorphism rs11614913 with decreased susceptibility to OA. Our results did not show an association between demographic, etiologic, and clinical characteristics with polymorphisms in TLR genes. However, we observed a significant association of the miR-196a2 rs11614913 polymorphism with sex, previous injury history, and a tendency to associate with higher BMI.

A large multi-centric study from 13 international cohorts stemming from nine populations identified TLR4 as an effector gene in OA, though another polymorphism rs10983775 in the TLR4 gene was reported as a risk variant for OA, and not the investigated polymorphisms rs4986790 and rs4986791 [21]. Studies of TLR4 gene D299G/T399I polymorphisms reported no association with RA susceptibility, an inflammatory disease that shares some symptoms with OA [23]. Currently, there are no data demonstrating that the analyzed functional polymorphism D299G/T399I in the TLR4 gene influences the expression of inflammatory cytokines in OA. However, our results are in line with previous findings demonstrating that investigated TLR4 polymorphisms are involved in impaired immune responses to viruses and TLR4 ligands such as lipopolysaccharide (LPS) and monophosphoryl lipid [24,25]. Functional polymorphisms in the TLR4 gene interfere with MyD88 recruitment and the expression of TRIF-dependent genes [26,27]. It has been shown that polymorphisms D299G/T399I are associated with increased expression of cytokines in inflammatory diseases such as chronic obstructive pulmonary disease [28] and Alzheimer’s disease [29], indicating that TLR4 polymorphisms that reduce the functionality of TLR4 may modify cytokine and chemokine profile, leading to chronic inflammation. Transfection of variant alleles D299G/T399I of the TLR4 gene into HEK initially causes a delay in the induction of the adaptive immune response upon LPS stimulation, but this reduction is accompanied by a constitutive increase in NF-κB and exaggerated immune reaction [27]. Thus, TLR4 polymorphisms D299G and T399I could delay NF-κB activation, leading to the increased expression of proinflammatory cytokines and altering the balance between the initiation of immune responses and excessive inflammation that leads to chronic inflammation. Given the functional influence of the investigated TLR4 polymorphisms, these genetic variations might predispose individuals to OA.

Our findings of an association of X-linked TLR7 rs3853839 polymorphism with an increased OA risk are in line with the findings of a recent study on knee OA [17] and in SLE [30]. The involvement of the TLR7 signaling pathway and TLR7 polymorphisms predisposing a patient to autoimmune and inflammatory diseases are particularly intriguing in those with sex-specific incidence, penetrance, and severity score of the phenotype.

Although our study did not show an association with OA risk, several studies have found a positive association between TLR9 SNP rs187084 and susceptibility to OA [31,32,33]. Previous studies reported the T allele of rs187084 as associated with advanced stages of knee OA [31] and hip OA [18], while another study reported the C allele as a risk factor for knee OA [33].

**Figure 1:**
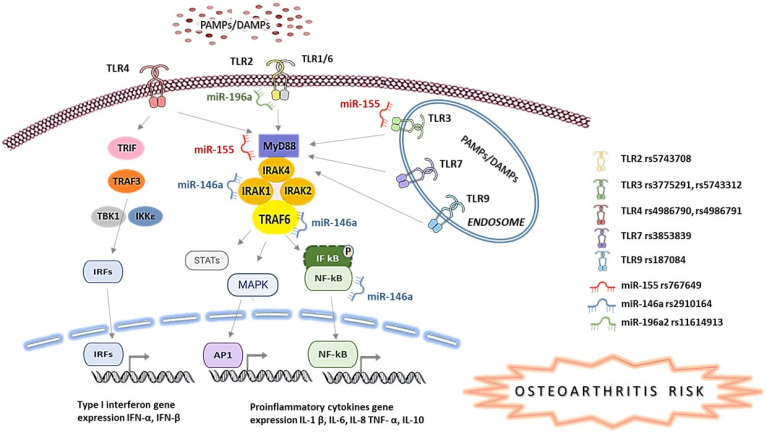
Schematic representation of the Toll-like receptor (TLR)-mediated signaling pathway. TLRs are controlled by several microRNAs, which, in addition to their role in the epigenetic regulation of gene expression, are ligands for TLR activation and downstream signaling. Genetic variations in the TLR and miRNA genes could influence cytokine production and susceptibility to osteoarthritis (OA). TLRs are indicated on the cell membrane (TLR4, TLR2, and TLR1/6) and endosomes (TLR3, TLR7, and TLR9) according to their distribution. Activation of the TLRs by PAMP/DAMP ligand binding initiates a complex signaling cascade. A MyD88-dependent signaling pathway activation results in the induction of NF-κB, and AP-1 and the production of pro-inflammatory cytokines (IL-1β, IL-6, and TNF-α). The TRIF proteins mediate the activation of the type I interferons gene expression (IFN-α and IFN-β). NF-κB—Nuclear factor kappa-light-chain-enhancer of activated B cells; AP-1—Activator protein 1; TRIF—TIR-domain-containing adapter-inducing interferon-β; TRAF3/6—TNF receptor-associated factor 3/6; TBK1—TANK-binding kinase 1; IKKε—Inhibitor of nuclear factor kappa-B kinase subunit ε; MyD88—Myeloid differentiation primary response 88; IRAK1/4—Interleukin-1 receptor-associated kinase 1/4; STATs—signal transducer and activator of transcription proteins; MAPK—mitogen-activated protein kinase; IRFs—interferon regulatory factors.

Cartilage deterioration in OA joints may be a consequence of TLR-ligand interactions with robust proinflammatory signaling [18]. TLR1, TLR2, TLR4, and TLR9 expression has been reported to be upregulated in OA chondrocytes [34], whereas TLR1-7 and TLR9 are upregulated in OA synovium [3]. A differential expression profile of TLRs is observed in osteoclastogenesis, with TLR4 and TLR9 predominantly expressed in mature osteoclasts [35]. Excessive/insufficient TLR activation leads to disruption of immune homeostasis and pro-catabolic TLR signaling in OA [36]. Upon recognition of DAMPs (HMGB1, RAGE, S100A9), TLRs trigger a network of interactions that induce the release of TNF-α and IL-1, the most potent cytokines that amplify the proinflammatory response leading to chronic inflammation [37]. Activation of NFkB, Activator protein 1 (AP-1), and IRF5 is tightly controlled by TLRs, as well as IRF3 and IRF7, which induce the expression of IFN type I gene and IFN-inducible genes [38], Figure 1.

Our study on the miR-196a2 polymorphism potentially identifies a novel variant associated with OA. Available studies on other inflammatory diseases found that miR-196a2 polymorphism rs11614913 shows an inverse association with chronic periodontitis [39], indicating its involvement in chronic inflammation. A pilot study demonstrated that the miR-196a rs11614913 variant allele is associated with type 1 diabetes risk; when compared to controls, patients had lower expression of miR-196a2 [9]. Although the same study found that miR-196a2 expression was not associated with different rs11614913 genotypes [9], these findings indicate that the decreased expression of miR-196a and its gene polymorphism rs11614913 could play an important role in the pathogenesis and susceptibility of this autoimmune disease. Another study found that miR-196a2 expression is significantly decreased in variant allele T carriers of the miR-196a2 rs11614913 polymorphism among patients with ulcerative colitis [40]. The miR-196a2 polymorphism rs11614913 was not associated with RA susceptibility in an Egyptian population [41] and Mexican population [42,43]. However, miR-196a2 rs11614913 C/T polymorphism shows an association with extra-articular manifestations in RA [43].

The role of different microRNAs polymorphisms in OA has not yet been comprehensively described. A number of miRNAs are involved in immune signaling pathways in OA, either in cartilage matrix degradation under inflammation or in protection against OA development [12]. Integrative molecular profiling of RNA-sequencing data and analysis of signaling pathways in OA revealed that miR-146a, miR-155, and miR-196a are differentially expressed between normal and OA cartilage [11]. A major influence on the regulation of immune mechanisms miRNAs could exert is through modulation of TLRs and cytokine receptor signaling [12]. Agonists of various TLRs induce the expression of specific miRNAs [44], but TLRs themselves can also be targeted directly by miRNAs that can bind to highly conserved target sites in the 3′ UTRs of these receptors [45]. A study on TLR1, TLR2, TLR4, and TLR6 knock-out animal models of OA implicates the TLRs and their regulating miRNAs in OA pathogenesis and disease progression [46].

miRs-196a2 is located within HOX gene clusters, between HOXC10 and HOXC9, and regulates HOX gene expression and transcription factors linked with the onset and development of OA [47]. In addition, miRs-196a regulates gene expression of ERG transcription factors, which have a role in articular cartilage endurance and resistance to osteoarthritic changes [48]. Increased expression of miR-196a decreases the proliferation of adipose-derived stem cells and enhances their osteogenic potential without affecting adipogenesis, which could potentially affect the chondrogenic potential of precursor stem cells in vitro [49]. The role of miR-196a2 in the potential deterioration of articular cartilage has been mainly studied in RA. The expression of innate immunity cytokines TNF-α and IL-1, as well as chemokines in monocytes, macrophages, and dendritic cells, were found to be under the control of miR-196a2. MiR-196a2 might have a major impact on innate immunity and TLR2 signaling by targeting NFƙB1 [7]. It has been demonstrated that the miR-196a expression profile in bone marrow-derived mesenchymal stem cells (BM-MSCs) can be altered by stimulation with the TLR2 agonist, PAM3CSK4, while miR-155 and miR-146a expression in BM-MSCs is upregulated upon stimulation with TLR4 agonist lipopolysaccharide (LPS) [50]. The miR-196a/TLR2 axis could be an attractive target for future research since the production of pro-inflammatory cytokines could potentially be manipulated by long-noncoding RNA (lncRNA) H19 [51].

Even though our study revealed no significant association between miR-146a and miR-155 polymorphisms and OA risk, numerous studies have shown aberrant expression of these miRNAs contributes to OA development and pathogenesis [13]. MiR-146a is abundantly expressed in early OA cartilage compared to normal tissue [52]. Furthermore, pro-inflammatory IL-1β and TNF-α are mediated by miR-146a [45], suggesting their potential role in chronic inflammation and degenerative changes in the osteoarthritic cartilage microenvironment. Upregulation of miR-146a increases apoptosis in human chondrocytes in response to mechanical injury [53], the most important risk factor in OA development. The biological effects of miR-146a are timely based on post-transcriptional repression of IRAK1 and TRAF6, downstream signaling molecules of most of the TLRs (TLR 2,4,5,7,8 and 9) [19,44]. Simultaneous overexpression of miR-146a and miR-140–5p has a strong protective effect against inflammatory cytokine production by targeting TLR4/NF-κB signaling in osteoarthritic chondrocytes [54]. It has been reported that miR-155 plays an important role in controlling inflammation by regulating TLR3 and TLR4 signaling [55]. Endogenous TLR ligands in the arthritic joint lead to upregulated expression of miR-155 in synovial monocytes and macrophages, triggering the increased production of the pro-inflammatory cytokines TNF-α, IL-1β and IL-6 [56] and the downregulation of SOCS1 and MyD88 [57] as well as anti-inflammatory SHIP-1 protein (SH2-containing inositol phosphatase-1), a TLR inhibitor that is also an important regulator of osteoblast proliferation and differentiation [58].

Targeting a single cytokine in OA and RA exhibited little effect. However, a broader approach targeting TLRs and/or microRNAs could show a higher upstream interference with OA inflammation. Although various TLR antagonists/inhibitors, from conventional small molecules to microRNA inhibitors and nano-drugs, have been investigated in preclinical studies for the treatment of inflammatory diseases and OA, only limited numbers of them have undergone clinical trials, and none have yet been approved for clinical use in OA [59,60], Recent studies and clinical trials investigated the use of the antimalarial drug hydroxychloroquine in the treatment of RA and SLE, identified to target endosomal TLR7/8/9 signaling as well as a number of small molecule inhibitors that target TLR2 and TLR4 [60].

Given the fact that various microRNAs are aberrantly expressed in OA, those findings open new possibilities for novel strategies in OA treatment. In an animal model of OA, a single intra-articular injection of either a miR-21 inhibitor or TLR7-9 antagonist induced long-lasting analgesia [61]. Genetic polymorphisms in TLRs and microRNA genes might potentially impede the effects of both TLR-targeted therapy and/or microRNA inhibitor therapy. Thus, a personalized approach and inclusion of TLR and microRNA genotyping data, combined with patient inflammatory status, might potentially help to identify more responsive patients and potentially improve the effectiveness of clinical trials and OA patients.

Development of disease-modifying therapies for osteoarthritis (DMOADs) and multiple biomarkers for improved patient stratification and applying the concepts of personalized and precision medicine, in combination with innovative and cell-based therapies, would provide a breakthrough to OA treatment in the future. Integration of a plethora of biological information, including genetics, epigenetics, genomics, and proteomics, combined with traditional clinical data, is needed for personalized/precision medicine and targeted therapy strategies. Artificial intelligence (AI) and machine learning have already been deployed to massive datasets in cancer [62] and early knee osteoarthritis diagnosis from X-ray images [63].

Moreover, another prospective role of miRNAs could be exerted through exosomes, small, 30–150 nm, phospholipid-bilayer-enclosed extracellular vesicles that carry numerous molecules as mediators of intercellular communication, including microRNAs. Since exosomes passively diffuse through tissues, they could be novel biomarkers in OA diagnosis and progression [60]. Furthermore, in vivo studies of the OA mouse model found that exosomes derived from fat mesenchymal stem cells protected cartilage and ameliorated OA symptoms via mTOR signaling inhibition [64]. These findings hold great potential in the regenerative medicine of OA.

The current study has several potential limitations that need to be addressed. First, the sample size of the current study was relatively small. Second, this study was performed at a single center and in a single Serbian population, which may limit the generalizability of the results to the Caucasian population. Although observed associations are encouraging, we examined a limited number of common polymorphisms, and other risk-related genetic variations in the examined genes as well as other TLR and miRNA genes that might influence susceptibility to OA should be investigated in the future. The data on TLRs as OA risk-conferring alleles vary across studies, which may be related to the etiology, demographic, and clinical heterogeneity of participants or the genetic background of the population. Despite these conflicting results, genetic changes in TLRs are widely recognized as important factors in immune system dysregulation that alter susceptibility to OA worldwide.

## 5. Conclusions

To conclude, OA has a complex genetic background and there is no single gene dominantly responsible for triggering the disease, but rather a complex network of multifunctional cellular mediators. Our findings indicate that polymorphisms in the TLR4 and TLR7 genes might increase OA risk and show a novel suggestive association of the miR-196a2 polymorphism rs11614913 variant allele with decreased susceptibility to OA. These findings may provide useful data for further research on the cross-talk between TLRs and miRNAs in OA. Further investigations are required to verify the potential role of TLR and microRNA gene polymorphisms and OA susceptibility in larger cohorts of patients.

## Figures and Tables

**Table 1:** Characteristics of genotyped TLR and miRNA polymorphisms.

Gene	Location	rs Number	SNP Change	Variant Type, Region	Amino Acid Change
**TLR2**	4q31.3	rs5743708	G2258A	Exon 3	Arg753Gln, R753Q
**TLR3**	4q35.1	rs3775291	G13909A	Exon 4	Leu412Phe, L412F
		rs5743312	C9948T	Intron 3	-
**TLR4**	9q33.1	rs4986790	A896G	Exon 3	Asp299Gly, D299G
		rs4986791	C1196T	Exon 4	Thr399Ile, T399I
**TLR7**	Xp22.2	rs3853839	C/G	3′ UTR	-
**TLR9**	3p21.2	rs187084	A/G	Promoter	-
**miR-196a2**	12q13.13	rs11614913	C/T	3′ UTR	-
**miR-146a**	5q33.3	rs2910164	C/G	Promoter	-
**miR-155**	21q21.3	rs767649	A/T	Promoter	-

**Table 2:** TLR gene variant associations with demographic and clinicopathologic features in osteoarthritis patients.

Demographic and Risk Factors		N	TLR2 rs5743708	TLR3 rs3775291	TLR3 rs5743312	TLR4 rs4986790	TLR4 rs4986791	TLR7 rs3853839	TLR9 rs187084
			GG/AG/AA	GG/GA/AA	CC/CT/TT	AA/AG/GG	CC/CT/TT	CC/CG/GG	AA/AG/GG
**Sex**	**Male**	35	26/8/1	14/20/1	21/14/0	26/9/0	12/20/3	26/0/9	10/17/8
	**Female**	60	40/20/0	21/36/3	39/19/2	44/16/0	24/35/1	36/6/18	21/25/14
	***p***		*0.256*	*0.810*	*0.428*	*1.000*	*0.259*	*0.115*	*0.771*
**Localization**	**Hip**	61	42/18/1	24/36/1	38/21/2	45/16/0	22/37/2	36/6/19	20/28/13
	**Knee**	34	24/10/0	11/20/3	22/12/0	25/9/0	14/18/2	26/0/8	11/14/9
	***p***		*0.753*	*0.227*	*0.566*	*1.000*	*0.695*	*0.090*	*0.834*
**Age median**	**<70**	46	33/13/0	17/26/3	29/16/1	33/13/0	19/25/2	29/3/14	16/18/12
	**>70**	49	33/15/1	18/30/1	31/17/1	37/12/0	17/30/2	33/3/13	15/24/10
	***p***		*0.592*	*0.543*	*0.999*	*0.816*	*0.790*	*0.905*	*0.613*
**BMI**	**≤25**	2	1/1/0	1/1/0	2/0/0	2/0/0	0/2/0	1/0/1	2/0/0
	**25–30**	77	52/24/1	27/47/3	50/25/2	56/21/0	30/43/4	53/5/19	26/31/20
	**>30**	16	13/3/0	7/8/1	8/8/0	12/4/0	6/10/0	8/1/7	3/11/2
	***p***		*0.793*	*0.920*	*0.516*	*0.682*	*0.655*	*0.566*	*0.072*
**Smoking**	**Yes**	17	12/4/1	6/10/1	12/5/0	15/2/0	4/13/0	10/2/5	7/5/5
	**No**	78	54/24/0	29/46/3	48/28/2	55/23/0	32/42/4	52/4/22	24/37/17
	***p***		*0.089*	*0.927*	*0.675*	*0.223*	*0.199*	*0.573*	*0.399*
**Physical activity**	**Yes**	20	17/3/0	8/11/1	15/5/0	17/3/0	5/13/2	14/0/6	7/8/5
	**No**	*75*	49/25/1	27/45/3	45/28/2	53/22/0	31/42/2	48/6/21	24/34/17
	***p***		*0.229*	0.917	0.409	0.259	0.187	0.425	0.913
**History** **of injury**	**Yes**	33	21/11/1	12/19/2	24/8/1	26/7/0	12/18/3	20/2/11	10/17/6
	**No**	62	45/17/0	23/37/2	36/25/1	44/18/0	24/37/1	42/4/16	21/25/16
	***p***		*0.304*	*0.806*	*0.281*	*0.471*	*0.224*	*0.740*	*0.539*
**Family history**	**No**	56	29/9/1	15/22/2	26/12/1	31/8/0	13/24/2	23/3/13	11/20/8
	**Yes**	39	37/19/0	20/34/2	34/21/1	39/17/0	23/31/2	39/3/14	20/22/14
	***p***		*0.275*	*0.882*	*0.781*	*0.347*	*0.723*	*0.560*	*0.510*
**Early** **OA onset**	**<55**	52	38/13/1	18/32/2	33/19/0	39/13/0	18/32/2	36/1/15	14/24/14
	**>55**	43	28/15/0	17/24/2	27/14/2	31/12/0	18/23/2	26/5/12	17/18/8
	***p***		*0.402*	*0.851*	*0.283*	*0.817*	*0.731*	*0.150*	*0.377*
**Menopause**	**Yes**	56	40/16/0	19/34/3	36/18/2	42/14/0	21/34/1	33/5/18	19/23/14
	**No**	4	0/4/0	2/2/0	3/1/0	2/2/0	3/1/0	3/1/0	2/2/0
	***p***		***0.003***	*0.755*	*0.874*	*0.275*	*0.333*	*0.300*	*0.510*
**Early menopause**	**Yes**	15	11/4/0	5/10/0	7/8/0	11/4/0	6/9/0	9/2/4	6/6/3
	**No**	45	29/16/0	16/26/3	32/11/2	33/12/0	18/26/1	27/4/14	15/19/11
	***p***		*0.753*	*0.559*	*0.097*	*1.000*	*0.842*	*0.862*	*0.882*
**Swelling**	**Yes**	32	22/10/0	10/19/3	20/12/0	23/9/0	13/17/2	24/0/8	11/12/9
	**No**	63	44/18/1	25/37/1	40/21/2	47/16/0	23/38/2	38/6/19	20/30/13
	***p***		*0.755*	*0.177*	*0.570*	*0.808*	*0.683*	*0.139*	*0.591*

**Table 3:** miRNA gene variant associations with demographic and clinicopathologic features in osteoarthritis patients.

Demographic and Risk Factors	Total N		miR-196a2 rs11614913	miR-146a rs2910164	miR-155 rs767649
			CC/CT/TT	GG/GC/CC	TT/TA/AA
**Sex**	**Male**	35	*11/13/11*	18/13/4	34/1/0
	**Female**	60	5/28/27	40/17/3	51/9/0
	***p***		***0.014***	*0.269*	*0.086*
**Localization**	**Hip**	61	13/26/22	38/19/4	55/6/0
	**Knee**	34	3/15/16	20/11/3	30/4/0
	***p***		*0.260*	*0.902*	*0.742*
**Age median**	**<70**	46	7/21/18	26/16/4	39/7/0
	**>70**	49	9/20/20	32/14/3	46/3/0
	***p***		*0.867*	*0.669*	*0.190*
**BMI**	**≤25**	2	0/0/2	1/0/1	2/0/0
	**25–30**	77	10/37/30	47/24/6	69/8/0
	**>30**	16	6/4/6	10/6/0	14/2/0
	***p***		*0.051*	*0.136*	*0.859*
**Smoking**	**Yes**	17	2/9/6	11/5/1	16/1/0
	**No**	78	14/32/32	47/25/6	69/9/0
	***p***		*0.641*	*0.933*	*0.684*
**Physical activity**	**Yes**	20	5/6/9	*11/7/2*	*19/1/0*
	**No**	75	*11/35/29*	*47/23/5*	*66/9/0*
	***p***		*0.337*	*0.785*	*0.683*
**History** **of injury**	**Yes**	33	10/9/14	18/11/4	30/3/0
	**No**	62	6/32/24	40/19/3	55/7/0
	***p***		***0.014***	*0.377*	*1.000*
**Family history**	**Yes**	56	9/14/16	23/12/4	36/3/0
	**No**	39	7/27/22	35/18/3	49/7/0
	***p***		*0.308*	*0.667*	*0.518*
**Early** **OA onset**	**<55**	52	9/22/21	30/18/4	47/5/0
	**>55**	43	7/19/17	28/12/3	38/5/0
	***p***		*0.981*	*0.754*	*0.751*
**Menopause**	**Yes**	56	4/26/26	38/15/3	47/9/0
	**No**	4	1/2/1	2/2/0	4/0/0
	***p***		*0.403*	*0.576*	*1.000*
**Early menopause**	**Yes**	15	0/8/7	10/5/0	10/5/0
	**No**	45	5/20/20	30/12/3	41/4/0
	***p***		*0.393*	*0.555*	***0.036***
**Swelling**	**Yes**	32	2/14/16	18/11/3	28/4/0
	**No**	63	14/27/22	40/19/4	57/6/0
	***p***		*0.109*	*0.754*	*0.728*

**Table 4:** Genotype frequencies and logistic regression analysis data for the TLR and miRNA gene polymorphisms in osteoarthritis.

TLRs Gene Variants	Genotype	Controls		OA Patients		*p* *	Adjusted OR ** [95% CI]	*p* ***
		N = 104	%	N = 95	*%*			
**TLR2** **rs5743708**	GG	71	68.27	66	69.47	*0.877*	0.930 [0.532–1.627]	*0.800*
	AG	31	29.81	28	29.47			
	AA	2	1.92	1	1.05			
**TLR3** **rs3775291**	GG	43	41.35	35	36.84	*0.400*	1.038 [0.644–1.674]	*0.878*
	GA	53	50.96	56	58.95			
	AA	8	7.69	4	4.21			
**TLR3** **rs5743312**	CC	78	75.00	60	63.16	*0.186*	1.587 [0.910–2.768]	*0.104*
	CT	24	23.08	33	34.74			
	TT	2	1.92	2	2.11			
**TLR4** **rs4986790**	AA	93	89.42	70	73.68	***0.004***	2.964 [1.364–6.442]	***0.006***
	AG	11	10.58	25	26.32			
	GG	0	0.00	0	0.00			
**TLR4** **rs4986791**	CC	90	86.54	36	37.89	***0.0001***	8.766 [4.435–17.328]	***0.00001***
	CG	13	12.50	55	57.89			
	GG	1	0.96	4	4.21			
**TLR7** **rs3853839**	CC	83	79.81	63	66.32	***0.033***	1.579 [1.106–2.255]	***0.012***
	CG	7	6.73	5	5.26			
	GG	14	13.46	27	28.42			
**TLR9** **rs1870840**	AA	35	33.65	31	32.63	*0.186*	1.253 [0.839–1.871]	*0.271*
	AG	55	52.88	42	44.21			
	GG	14	13.46	22	23.16			
**miR-196a2** **rs11614913**	CC	4	3.85	16	16.84	***0.010***	0.619 [0.397–0.964]	***0.034***
	CT	52	50.00	41	43.16			
	TT	48	46.15	38	40.00			
**miR-146a** **rs2910164**	GG	68	65.38	58	61.05	*0.344*	1.328 [0.821–2.146]	*0.248*
	GC	33	31.73	30	31.58			
	CC	3	2.88	7	7.37			
**miR-155** **rs767649**	TT	94	90.38	85	89.47	*0.831*	1.119 [0.439–2.850]	*0.813*
	TA	10	9.62	10	10.53			
	AA	0	0	0	0			

*: *p* values for assessment of genotype frequencies among OA cases and controls, by χ^2^ or Fisher exact test; **: odds ratio (OR) adjusted for sex and age; ***: *p* values for adjusted OR via logistic regression analysis.

## Data Availability

Not applicable.
